# Surgical Site Infection Following Single-Port Appendectomy: A Systematic Review of the Literature and Meta-Analysis

**DOI:** 10.3389/fsurg.2022.919744

**Published:** 2022-06-08

**Authors:** Franziska Köhler, Lena Reese, Carolin Kastner, Anne Hendricks, Sophie Müller, Johan F. Lock, Christoph-Thomas Germer, Armin Wiegering

**Affiliations:** ^1^ Department of General, Visceral, Transplantation, Vascular and Pediatric Surgery, University Hospital, University of Wuerzburg, Wuerzburg, Germany; ^2^ Department of Biochemistry and Molecular Biology, University of Wuerzburg, Wuerzburg, Germany; ^3^ Comprehensive Cancer Center Mainfranken, University of Wuerzburg Medical Centre, Wuerzburg, Germany

**Keywords:** appendicitis, appendectomy, surgical site infection, single-port appendectomy, conventional laparoscopic appendectomy, wound infection, SSI

## Abstract

**Introduction:**

Surgical site infections (SSIs) are one of the most common postoperative complications after appendectomy leading to recurrent surgery, prolonged hospital stay, and the use of antibiotics. Numerous studies and meta-analyses have been published on the effect of open versus conventional laparoscopic appendectomy (CLA) reporting faster postoperative recovery and less postoperative pain for CLA. A development from CLA has been the single-port appendectomy (SPA), associated with a better cosmesis but seemingly having a higher risk of wound infections. The aim of this systematic literature review and meta-analysis is to investigate whether reduced port or SPA alters the ratio of SSIs.

**Methods:**

Pubmed, Embase, and Cochrane databases were screened for suitable articles. All articles published between January 1, 2002, and March 23, 2022, were included. Articles regarding children below the age of 18 were excluded as well as manuscripts that investigated solemnly open appendectomies. Articles were screened for inclusion criteria by two independent authors. Incidence of SSI was the primary outcome. Duration of operation and length of hospital stay were defined as secondary outcomes.

**Results:**

A total of 25 studies were found through a database search describing 5484 patients. A total of 2749 patients received SPA and 2735 received CLA. There was no statistical difference in the rate of SSI (*P* = 0.98). A total of 22 studies including 4699 patients reported the duration of operation (2223 SPA and 2476 CLA). There was a significantly shorter operation time seen in CLA. The length of hospital stay was reported in 23 studies (4735 patients: 2235 SPA and 2500 CLA). A shorter hospital stay was seen in the SPA group (*P* < 0.00001). Separately performed analysis of randomized controlled trials could not confirm this effect (*P* = 0.29).

**Discussion:**

SPA is an equally safe procedure considering SSI compared to CLA and does not lead to an increased risk of SSI. A longer operation time for SPA and a minor difference in the length of stay does lead to the use of SPA in selected patients only.

## Introduction

Acute appendicitis (AA) is one of the most common causes of acute abdominal pain and the most frequent indication of abdominal emergency surgery worldwide (1, 2). AA can be divided into uncomplicated appendicitis i.e., phlegmonous and complicated appendicitis including perforation, abscess, and peritonitis (2).

The current gold standard treatment is appendectomy, in the majority of cases performed laparoscopically. However, antibiotic therapy seems to be an alternative in uncomplicated cases (3–7). In recent years, single-port appendectomy (SPA) using only one incision in or below the umbilicus has become more and more popular (8). It is thought to provide better wound cosmesis and faster recovery compared to conventional laparoscopic appendectomy (CLA) (9, 10). SPA can be performed in different techniques, first, by using designated single ports that have been developed for single-port laparoscopy. These trocars provide three single channels through which the instruments are inserted (11). Second, three conventional trocars can be inserted in or below the umbilicus (12). With this technique, it is important to incision the fascia sparingly and insert each trocar through its own fascial incision to reduce gas efflux (13). Third, self-made single ports have been established using rings, bands, and surgical gloves (14).

Appendectomy, performed open or laparoscopically, are surgical procedures with manageable perioperative risk and low mortality (15). Bleeding, stump insufficiency, or intraabdominal abscess are rather rare complications (16). Surgical site infections (SSIs) appear in up to 9% of appendectomies and therefore present the most frequent complication after appendectomy (15, 17).

According to the Center of Disease Control (CDC), SSI can be divided into superficial incisional surgical site infection, deep incisional site infection, and organ/space surgical site infection (see Table 1) (18, 19).

**Table 1:** Classification of surgical site infection according to the CDC (Center of Disease Control) (11, 12).

Surgical site infection	Criteria
Superficial incisional surgical site infection	Occurs within 30 days after surgery; involves only the skin and subcutaneous tissue of the incision
Deep incisional surgical site infection	Occurs within 30 or 90 days after surgery; involves deep soft tissues of the incision (muscle and fascial layers)
Organ/space surgical site infection	Occurs within 30 or 90 days after surgery; involves tissue deeper than fascial/muscle layers that have been opened or manipulated during the surgery

The aim of this study was to evaluate the influence of SPA on the occurrence of superficial incisional and deep incisional surgical site infection compared to CLA.

## Methods

### Study Selection and Search Strategy

PubMed database, Embase database, and Cochrane database were searched on March 23, 2022. Search terms were append* and *SSI* or *surgical site infection* or *local infection.* Studies with available full text in English or German language were included in the analysis. No study type was excluded. Manuscripts that focused on pediatric patients (below the age of 18) were excluded. Outcomes of interest were defined and are listed in Table 2 with the primary outcome being the incidence of SSI.

**Table 2:** Table of primary and secondary outcomes of interest and inclusion and exclusion criteria.

**Primary outcome of interest**	**Secondary outcome of interest**
Incidence of surgical site infection (SSI)	Length of hospital stay in days
	Operation time in minutes
**Inclusion criteria**	**Exclusion criteria**
Studies published between January 1, 2002 and March 23, 2022 reporting the incidence of SSI	Studies focusing on patients below the age of 18

Duplicates were removed and articles were first screened by title and abstract and second reviewed in full text for eligibility criteria by two independent reviewers (FK and LR). Disagreement on the eligibility of articles was discussed and solved by consensus.

Additionally, studies used in preexisting meta-analysis were screened and included if full-text screening did not reveal exclusion criteria.

**Figure 1:**
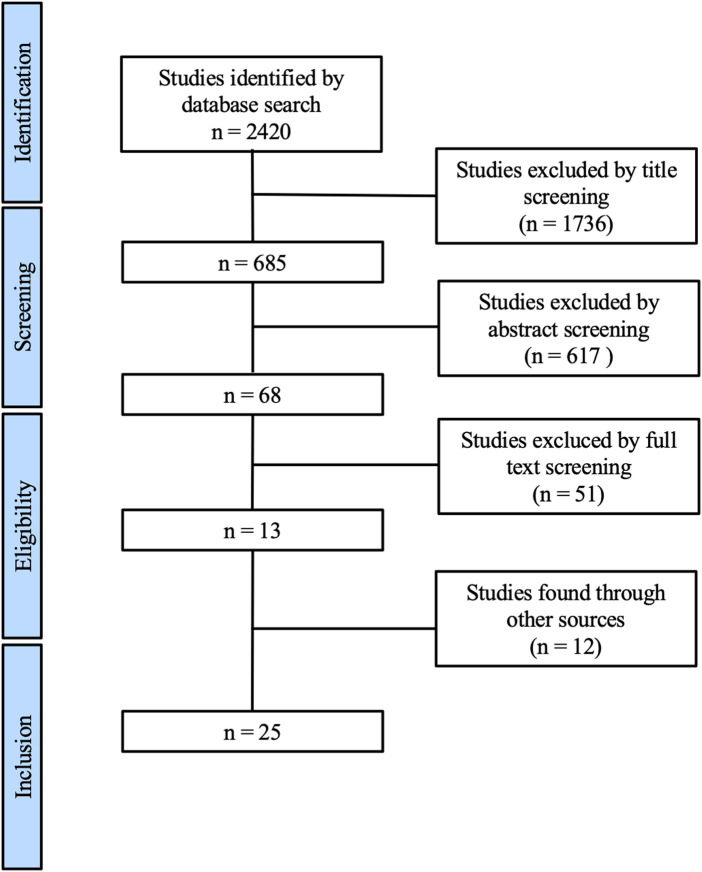
Preferred Reporting Items for Systematic Reviews and Meta-Analysis flow diagram of the study identification and selection process (13).

The systematic review and meta-analysis were performed in line with the PRISMA (Preferred Reporting Items for Systematic Reviews and Meta-Analysis) guidelines. The study selection process is pictured in the PRISMA flowchart (Figure 1) (20).

Literature organization was performed using program EndNoteX9, while charts, tables, and statistical analysis were obtained using RevMan5, Prism Graphpad, Microsoft Word, and PowerPoint. The measure of effects was assessed with the odds ratio (OR) and fixed effects model as well as the corresponding 95% confidence interval (CI 95%). Statistical significance was assessed by performing descriptive statistics. Statistical heterogeneity was assessed by calculating the *χ*^2^ and *I*^2^ tests.

### Risk of Bias Assessment

The risk of bias was assessed using the ROBINS-I tool for uncontrolled before-after studies (21), as the minority of studies were randomized controlled trials. Evaluated risks of bias were as follows: bias due to confounding, in the selection of participants in the study, in the classification of intervention, due to deviations from intended interventions, due to missing data, in the measurement of outcome, and in the selection of the reported result as well as the overall risk of bias.

The risk of bias was divided into low, medium, and high risk of bias as well as unclear risk of bias if no information regarding the evaluated risk of bias was available in the study. Detailed risk of bias is listed in Table A1 in the Appendix.

The overall risk of bias assessment revealed a low risk, even in the non-randomized controlled trials.

## Results

After removing duplicates, a literature search revealed 2420 studies. Through title and abstract screening, 68 manuscripts were found to be suitable for full-text screening. A total of 13 studies meet the inclusion criteria. Throughout the literature search additionally six meta-analyses were found. By screening the literature that was used to perform these meta-analyses, 12 further studies were identified that met the inclusion criteria. Overall 25 manuscripts were included in the meta-analysis (see Figure 1).

### Primary Outcome

The primary outcome was defined as the incidence of SSI. A total of 25 studies were identified that investigated the effect of single-port or reduced-port appendectomy on the incidence of SSI (11–13, 22–44). In two studies (35, 37) both groups did not report any SSIs, therefore OR was not estimable. Overall 5484 patients were included in the analysis. A total of 2749 patients received SPA and 2735 patients CLA. Of the patients treated with SPA 104 developed SSI and 110 patients developed SSI in the CLA group. There was no significant difference in the two groups estimable (*P* = 0.98) (see Supplementary Figures S2 and S3).

Furthermore, randomized controlled trials were investigated separately. Nine trials were identified through database search (11, 12, 25, 27, 30, 34, 36, 37). The trial by Carter et al. reported no SSIs in both study groups, therefore OR was not estimable (37). Overall 1143 patients were included in the analysis, 554 received SPA and 589 received CLA. A total of 72 patients developed SSI, 27 in the single-port group and 45 in the conventional group. No statistically significant difference was seen between the groups (*P* = 0.06) (see Supplementary Figures S4 and S5).

### Secondary Outcome

#### Operation Time

Out of the studies that reported the incidence of SSI, 22 studies reported the duration of the performed surgery. Overall 4699 patients were included in the analysis on surgery time, 2223 received SPA and 2476 received CLA. One study did not report the standard deviation; therefore, OR was not estimable (22). There was a significant difference between the two groups with shorter operation time in the CLA group (*P* < 0.00001) (see Supplementary Figures S6 and S7).

The mean operation time was 53.52 min (SD 13.65) for SPA and 50.83 min (SD 15.75) for CLA.

Looking at randomized controlled trials only, 8 trials were identified that included 931 patients, 465 in the SPA group and 466 in the laparoscopic group. In line with the results of the analysis of all studies, there was a significantly longer surgery time in the single-port group (*P* < 0.00001) (see Supplementary Figures S8 and S9). The mean operation time was 55.67 min (SD 19.45) in the single-port group and 51.81 min (SD 23.06) in the CLA group.

#### Hospital Stay

Out of the studies that reported SSI in SPA and CLA, 23 investigated the length of hospital stay. 4735 patients were included in the analysis, 2235 in the single-port group and 2500 in the CLA group. In five studies, information was missing to perform further analysis (22, 27, 28, 38, 41). There was a significant difference between the two groups (*P* < 0.00001) favoring SPA (see Supplementary Figures S10 and S11). The mean length of stay was 2.93 days (SD 1.28) in the single-port group and 3.05 days (SD 1.17) in the CLA group.

Looking at only randomized controlled trials, there were eight studies found through a database search. Two studies did not provide enough information to perform further analysis (27, 28). Overall 852 patients were analyzed, 428 in the SPA group and 424 in the CLA group. There was no statistical significance in the two groups (*P* = 0.29) (see Supplementary Figures S12 and S13). The mean length of stay was 2.64 days (SD 0.92) in the single-port group and 2.6 days (SD 0.87) in the CLA group.

## Discussion

This systematic literature review and meta-analysis revealed no difference in the incidence of SSI for single-port appendectomy compared to CLA. Operation time was significantly shorter in the CLA group, while hospital length was significantly shorter in the SPA group.

On one hand, the updated guideline of the World Society of Emergency Surgery (WSES) on diagnosis and treatment of AA claims that SPA is equally safe and effective as CLA. On the other hand, the listed study in the guideline revealed longer operation time, higher rates of wound infection, and requirement for higher doses of pain medication while SPA does provide better wound cosmesis. Overall, the updated guideline does not recommend SPA over CLA due to the listed disadvantages (45). This meta-analysis did not investigate the use of pain medication, while first it can confirm longer operation time and second it did not show higher rates of SSI in the SPA group (46). Longer operation times and higher doses of pain medication (while the postoperative pain level did not reveal any difference) are socioeconomic factors that should not be the only aspects to be considered when deciding on one or the other procedure.

Duration of surgery varied broadly between the different studies, with means ranging from 32.6 to 84.8 min for SPA and 29.5 to 89 min in the CLA group. The difference between the means of the two groups is estimated at 3 min. When looking at the studies that had more than 100 patients in every group (23, 28, 42, 47), all of them were single-center studies and surgeries mostly performed by one surgeon. Operation time in these studies ranged from 34 to 43.8 in the SPA group and 29.8 to 42.28 min in the CLA group, which is a shorter duration than the median operation time if looking at all study types. Studies have revealed lower mortality for abdominal surgical procedures in high-volume centers (48) and furthermore a learning curve for laparoscopic skills (49). Therefore, it is likely that surgeons performing higher numbers of appendectomies (SPA and CLA) are able to do these procedures in a shorter duration. This should be considered when deciding between the two surgical procedures, as otherwise this review and meta-analysis were not able to reveal additional disadvantages for SPA compared to CLA and even show a shorter hospital stay for SPA.

A literature search revealed more than 5000 patients to be included through 25 studies in this analysis, which leads to one of the largest meta-analysis on this topic to date. Analyzation of randomized controlled trials and all studies did reveal matching results, except for the length of hospital stay in the overall analysis. Looking at only randomized controlled trials, which did not reveal a difference between SPA and CLA regarding the length of stay, the results of this meta-analysis are in line with the existing meta-analysis (9, 10).

Surgical techniques and instruments used in the studies included in the meta-analysis varied broadly, reaching from self-made incisional ports using surgical gloves to designated single-port trocars. This might be a risk of bias, as the procedure in itself varies and makes comparability difficult. The reason for the use of self-made single ports is mainly the higher costs of manufactured single-port trocars as well as availability in low-income countries (29). Studies investigating the self-made incisional ports reported a low complication rate and good postoperative cosmesis results (23, 43). However, there is still a lack of studies comparing self-made single ports with manufactured single-ports. Especially randomized controlled trials focusing on cost-effectiveness and long-term outcomes are missing. Furthermore, contrary to the suspicion that SPA is associated with higher costs, the study by Goodman et al. revealed no difference in costs between SPA and CLA and Wieck et al. even reported significantly lower costs in the SPA group (50, 51).

In a high-quality meta-analysis by Zaman et al. who solemnly analyzed randomized controlled trials (and included pediatric patients in their analysis), a higher cosmetic score in the SPA group was reported (52). We did not analyze the cosmetic aspect in our analysis on SPA versus CLA, but it seems likely that one incision compared to three incisions results in a better cosmetic score.

This analysis has some limitations. First, all study types were included in the analysis. Therefore, it might be possible that low-quality studies were included in the analysis, which might affect the overall validity of this analysis, so we also performed an analysis on only randomized controlled trials that were found through the literature search. The analysis of randomized controlled trials alone included more than 1400 patients and the results are in line with the ones of the overall analysis except for the length of stay. On the other hand, the risk of bias assessment for all studies revealed rather high quality and low risk of bias for all studies (see Table A1 in the appendix).

The influence of the surgical approach on hospital length of stay does show a statistical significance between the SPA and CLA groups. Nevertheless, the difference does add up to merely 3 h (171 min). Overall, this difference does not seem to be of clinical importance, as most patients are discharged after morning rounds, regardless if surgery took place in the morning or in the afternoon.

The aim of this analysis was to investigate only superficial and deep incisional surgical site infection and exclude deep/organ space infection. A number of studies divided SSI into superficial, deep, and organ/space according to the CDC classification. Some studies reported “wound infection” without further clarification. Therefore, it might be possible that to some extent deep SSIs are included in the analysis and distort the results.

Looking at the length of hospital stay, a limitation might be, that not all studies reported the overall hospital stay but described the postoperative hospital stay instead. We analyzed “postoperative hospital stay” and “hospital stay” under the same category. This might be an explanation for the differing results when analyzing all study types and randomized controlled trials separately and needs to be considered when interpreting the data.

## Conclusion

SPA seems to be a safe alternative to CLA with equal risk for wound infection. It needs to be considered that SPA takes significantly longer operation time but leads to significantly shorter hospital length of stay, even if the latter is of questionable clinical importance.

## Figures and Tables

**Table A1:** Assessment of risk of bias using the ROBINS-I Tool (Risk of Bias in Non-randomized Studies of Intervention).

	Baseline confounding	Selection of participants	Classification of intervention	Deviation from intended intervention	Missing data	Measurement of outcomes	Selection of reported results	Overall risk of bias
Ahmed et al. (11)	Low	Low	Low	Low–moderate	Low–moderate	Low	Low	Low
Amos et al. (32)	Low	Moderate	Low	Low	Low	Low	Low	Low
Carter et al. (37)	Low	Low	Low	Low	Low	Low	Low	Low
Ceci et al. (38)	Moderate	High	Low	n/a	n/a	Low	Moderate	Moderate
Cho et al. (39)	Moderate	n/a	Low	n/a	Low	Low	Low	Low–moderate
Choi et al. (40)	Low	Low	Low	Moderate	Low	Low	Low	Low–moderate
Chow et al. (13)	Low	Low	Low	n/a	Low	Low	Low	Low
Donmez et al. (41)	Moderate	Moderate	Low	n/a	Low	Low	Low	Moderate
Fatima-Tu-Zahara et al. (36)	Low	Low	Low	Low	Low	Low	Low	Low
Jategaonkar et al. (42)	Low	Low	Low	n/a	Low	Low	Low	Low
Kang et al. (43)	Low	n/a	Low	Moderate	Low	Low	Low	Low–moderate
Kim et al. (22)	Low	Low	Moderate	Low	Low	Low	Low	Low–moderate
Kim et al. (23)	Low	Low	Low	Low	Low	Low	Low	Low
Kye et al. (25)	Low	Low	Low	Low	Low	Low	Low	Low
Lee et al. (26)	Low	Low	Low	Low	Low	Low	Low	Low
Lee et al. (44)	Low	Moderate	Low	Low	Low	Low	Low	Low–moderate
Lee et al. (28)	Low	Low	Low	Low	Low	Low	Low	Low
Lee et al. (29)	Low	Moderate	Low	Moderate	Low	Low	Low	Moderate
Pan et al. (30)	Low	Low	Low	Low	Low	Low	Low	Low
Park et al. (27)	Low	Low	Low	Low	Low	Low	Low	Low
Park et al. (31)	Low	Low	Low	Low	Low	Low	Low	Low
Raakow et al. (33)	Low	Moderate	Low	Low	Low	Low	Low	Low–moderate
Sozutek et al. (34)	Low	Low	Low	Low	Low	Low	Low	Low
Teoh et al. (12)	Low	Low	Low	Low	Low	Low	Low	Low
Vidal et al. (35)	Low	Low	Low	Low	Low	Low	Low	Low

*Low risk of bias, the study is comparable to a well-performed randomized trail; moderate risk of bias, the study appears to provide sound evidence for a non-randomized study but cannot be considered comparable to a well-performed randomized trial; high of bias, the study has some important problems; unclear risk, no information.*

## Data Availability

The original contributions presented in the study are included in the article/Supplementary Material, further inquiries can be directed to the corresponding author/s.
