# Supplementary figures and images for: Surgical Site Infection Following Single-Port Appendectomy: A Systematic Review of the Literature and Meta-Analysis
Source: Front Surg. 2022 Jun 8;9:919744. doi: 10.3389/fsurg.2022.919744 (PMC9213668; doi:10.3389/fsurg.2022.919744)

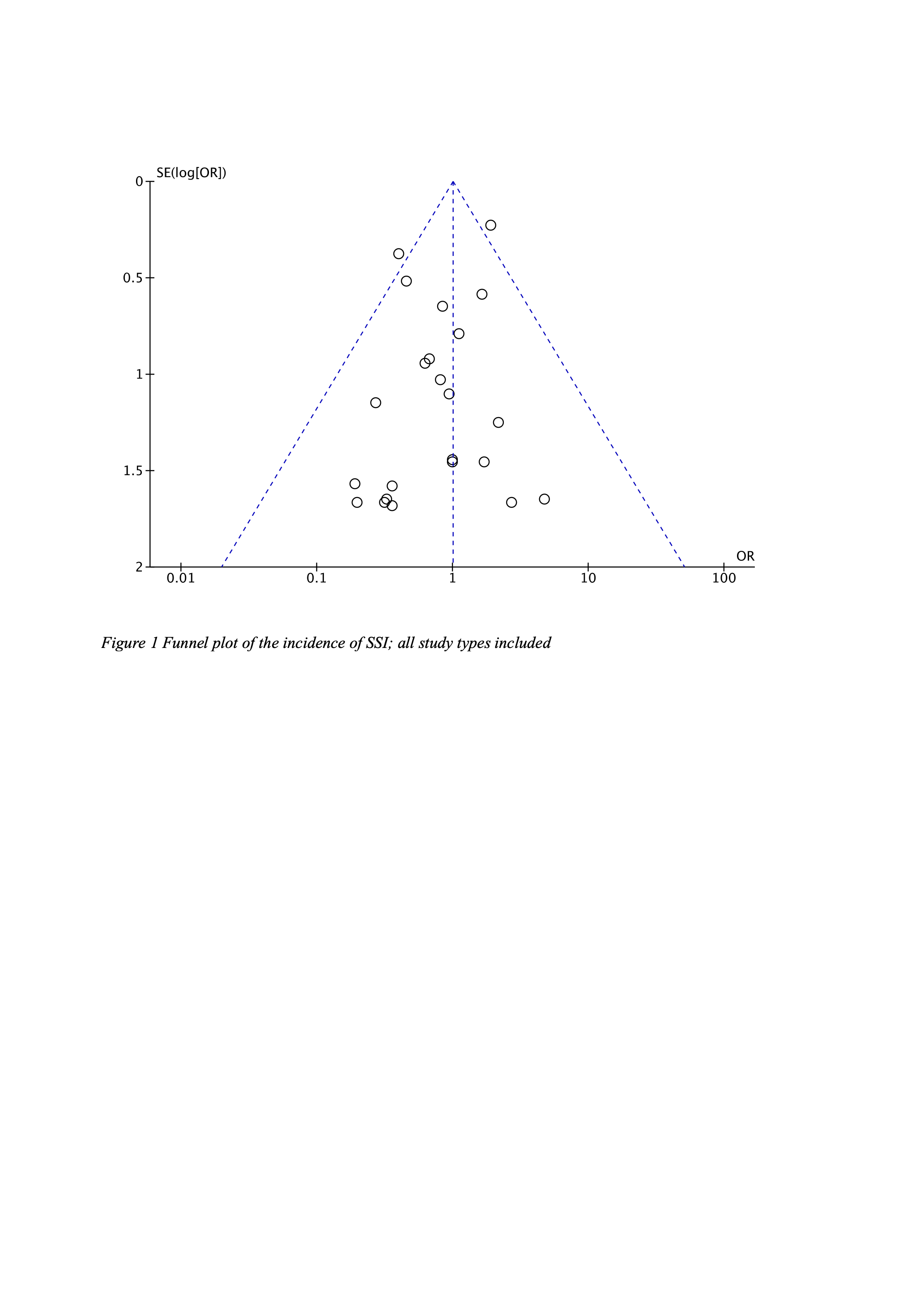

Supplement: Supplementary file 1 [file Image_1_v1.tiff]

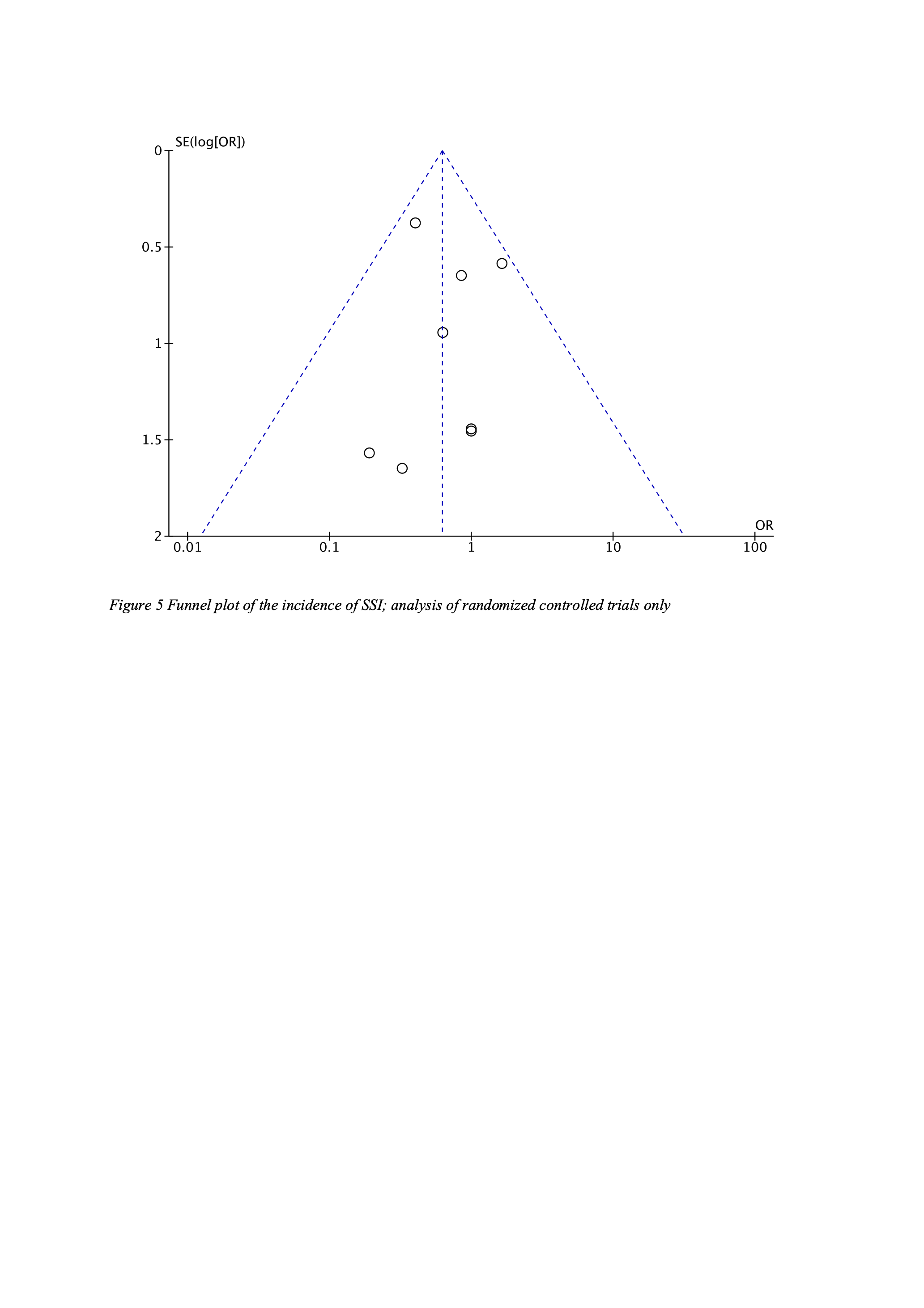

Supplement: Supplementary file 2 [file Image_2_v1.tiff]

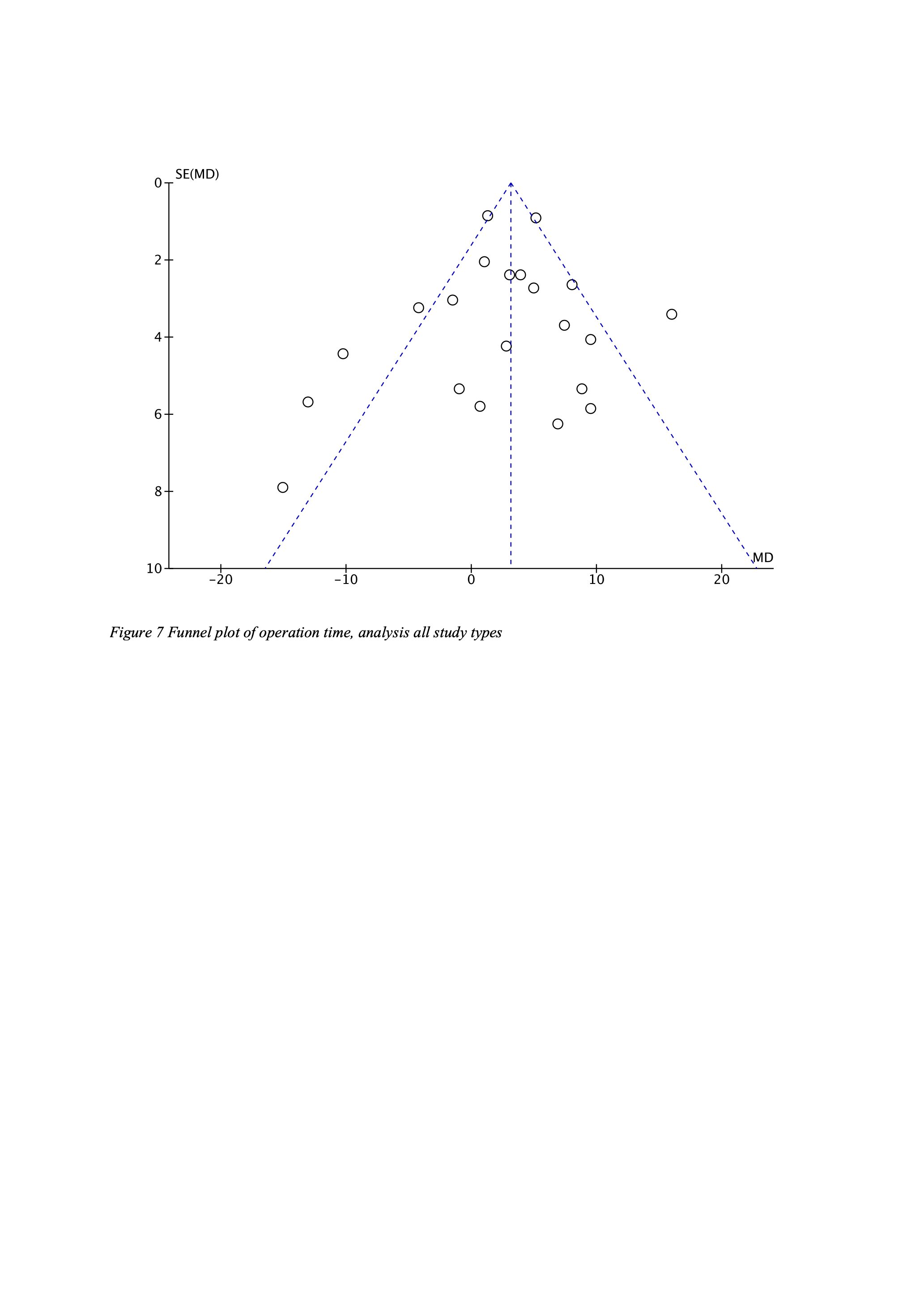

Supplement: Supplementary file 3 [file Image_3_v1.tiff]

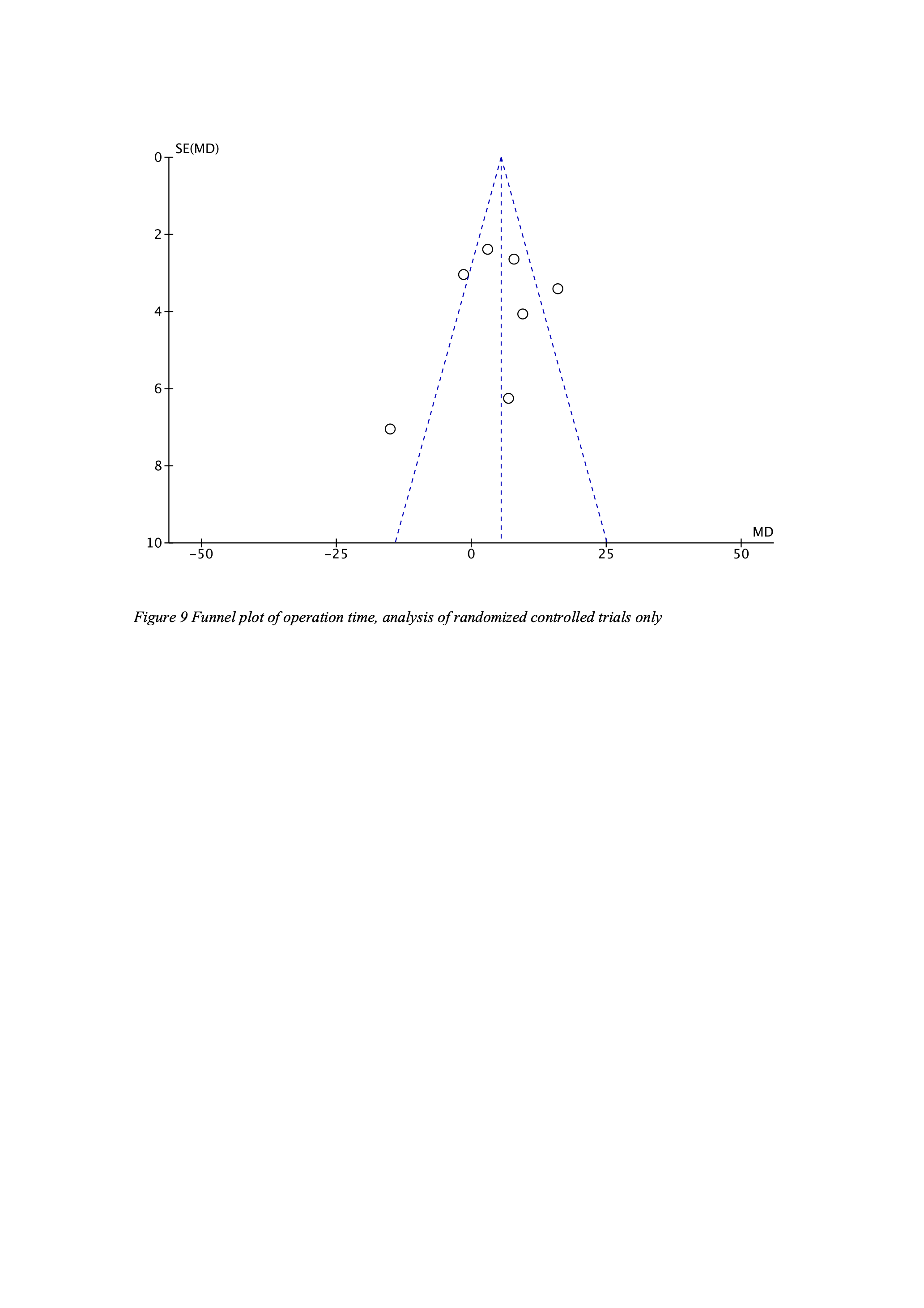

Supplement: Supplementary file 4 [file Image_4_v1.tiff]

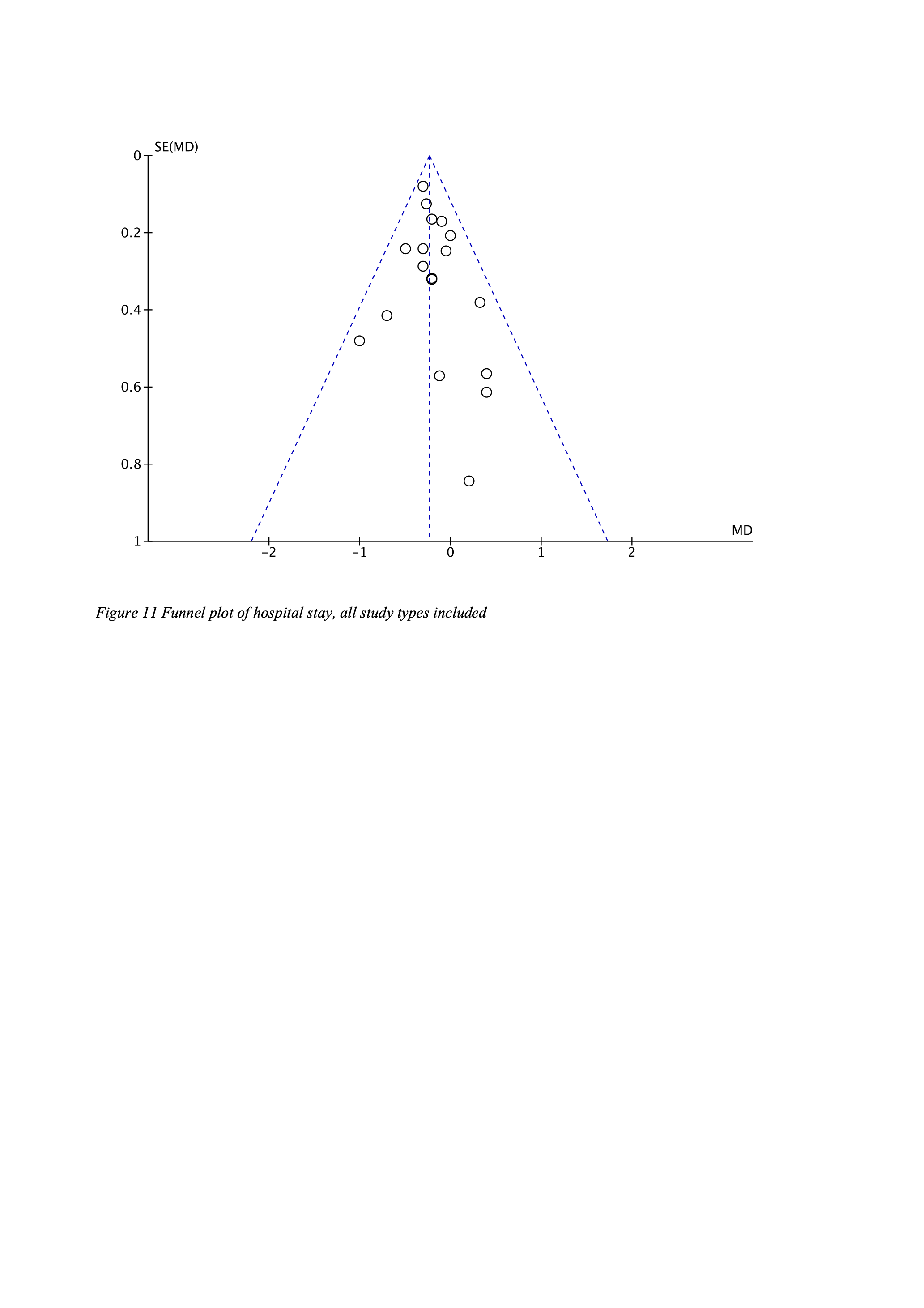

Supplement: Supplementary file 5 [file Image_5_v1.tiff]

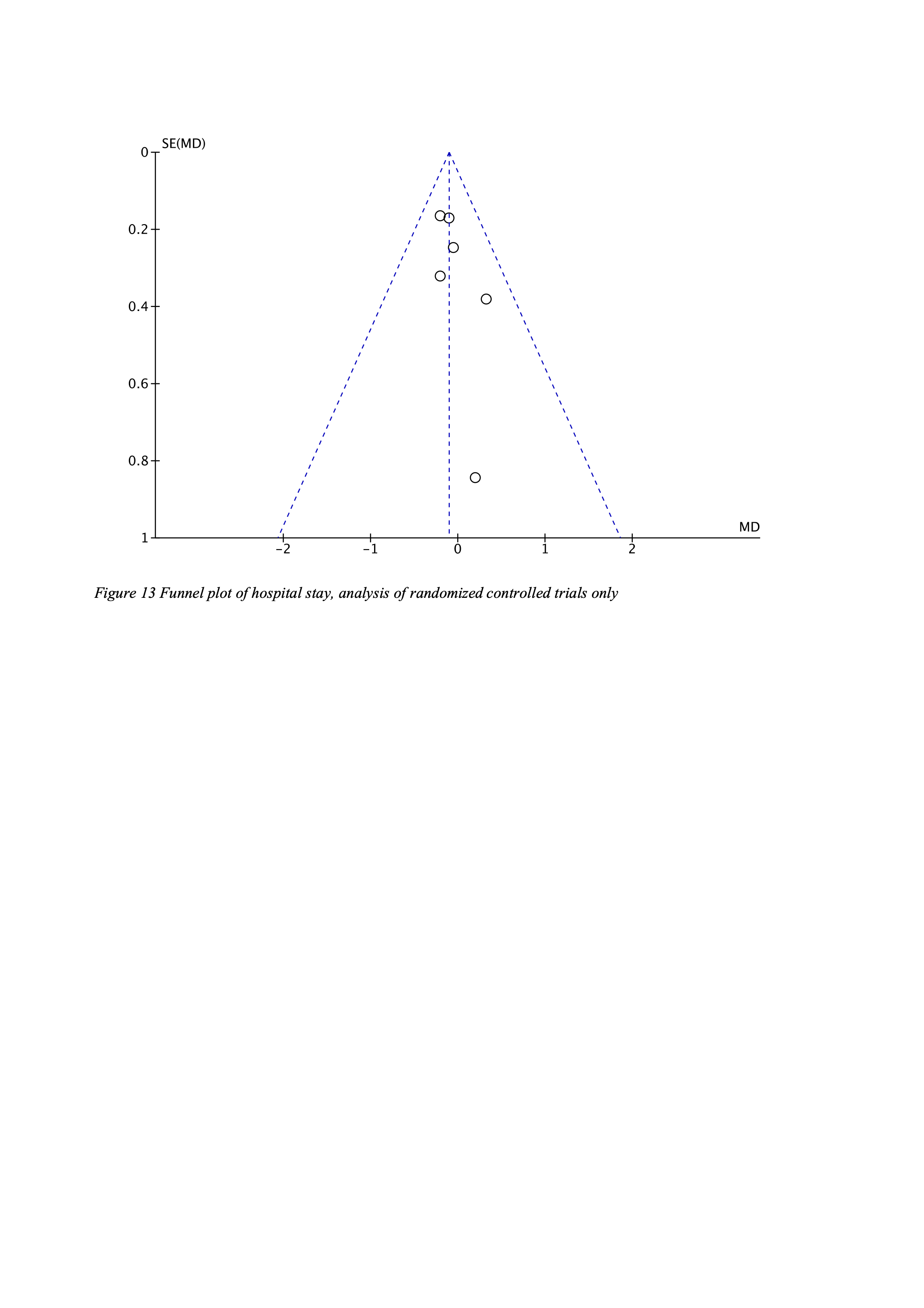

Supplement: Supplementary file 6 [file Image_6_v1.tiff]
