# Supplementary material for: Surgical Site Infection Following Single-Port Appendectomy: A Systematic Review of the Literature and Meta-Analysis
Source: Front Surg. 2022 Jun 8;9:919744. doi: 10.3389/fsurg.2022.919744 (PMC9213668; doi:10.3389/fsurg.2022.919744)
Supplement: Supplementary file 7 [file Table_1_(3).docx]

| **Surgical site infection:** | **Criteria:** |
| --- | --- |
| superficial incisional surgical site infection | occurs within 30 days after surgery;  involves only the skin and subcutaneous tissue of incision |
| deep incisional surgical site infection | occurs within 30 or 90 days after surgery;  involves deep soft tissues of the incision (muscle and fascial layers) |
| organ/ space surgical site infection | occurs within 30 or 90 days after surgery;  involves tissue deeper than fascial/ muscle layers that has been opened or manipulated during the surgery |

Table 1 Classification of surgical site infection according to the CDC (Center of Disease Control) (11, 12)
