# Supplementary material for: Surgical Site Infection Following Single-Port Appendectomy: A Systematic Review of the Literature and Meta-Analysis
Source: Front Surg. 2022 Jun 8;9:919744. doi: 10.3389/fsurg.2022.919744 (PMC9213668; doi:10.3389/fsurg.2022.919744)
Supplement: Supplementary file 8 [file Table_1_v1.docx]

|  | Baseline confounding | Selction of participants | Classification of intervention | Deviation from intended intervention | Missing data | Measurement of outcomes | Selection of reported results | Overall risk of bias |
| --- | --- | --- | --- | --- | --- | --- | --- | --- |
| Ahmed et al.  2015 | low | low | low | low-moderate | low-moderate | low | low | low |
| Amos et al.  2012 | low | moderate | low | low | low | low | low | low |
| Carter et al.  2014 | low | low | low | low | low | low | low | low |
| Ceci et al.  2013 | moderate | high | low | n/a | n/a | low | moderate | moderate |
| Cho et al.  2011 | moderate | n/a | low | n/a | low | low | low | low - moderate |
| Choi et al.  2019 | low | low | low | moderate | low | low | low | low-moderate |
| Chow et al.  2010 | low | low | low | n/a | low | low | low | low |
| Donmez et al. 2016 | moderate | moderate | low | n/a | low | low | low | moderate |
| Fatima-Tu-Zahara et al. 2020 | low | low | low | low | low | low | low | low |
| Jategaonkar et al. 2014 | low | low | low | n/a | low | low | low | low |
| Kang et al.  2010 | low | n/a | low | moderate | low | low | low | low-moderate |
| Kim et al.  2012 | low | low | moderate | low | low | low | low | low-moderate |
| Kim et al.  2015 | low | low | low | low | low | low | low | low |
| Kye et al.  2013 | low | low | low | low | low | low | low | low |
| Lee et al.  2009 | low | low | low | low | low | low | low | low |
| Lee et al.  2010 | low | moderate | low | low | low | low | low | low-moderate |
| Lee et al.  2013 | low | low | low | low | low | low | low | low |
| Lee et al.  2016 | low | moderate | low | moderate | low | low | low | moderate |
| Pan et al.  2013 | low | low | low | low | low | low | low | low |
| Park et al.  2010 | low | low | low | low | low | low | low | low |
| Park et al.  2012 | low | low | low | low | low | low | low | low |
| Raakow et al. 2011 | low | moderate | low | low | low | low | low | low-moderate |
| Sozutek et al. 2013 | low | low | low | low | low | low | low | low |
| Teoh et al.  2012 | low | low | low | low | low | low | low | low |
| Vidal et al.  2010 | low | low | low | low | low | low | low | low |

Low risk of bias = the study is comparable to a well-performed randomized trail; moderate risk of bias = the study appears to provide sound evidence for a non- randomized study but cannot be considered comparable to a well-performed randomized trial; high of bias = the study has some important problems; unclear risk = no information

Table 3: Assesment of risk of bias using the ROBINS-I Tool (Risk Of Bias in Non-randomized Studies of Intervention)
