# Supplementary material for: Surgical Site Infection Following Single-Port Appendectomy: A Systematic Review of the Literature and Meta-Analysis
Source: Front Surg. 2022 Jun 8;9:919744. doi: 10.3389/fsurg.2022.919744 (PMC9213668; doi:10.3389/fsurg.2022.919744)
Supplement: Supplementary file 9 [file Table_2_(1).docx]

| **Primary Outcome of Interest** | **Secondary Outcome of Interest** |
| --- | --- |
| Incidence of surgical site infection (SSI) | Length of hospital stay in days |
|  | Operation time in minutes |
| **Inclusion Criteria** | **Exclusion Criteria** |
| Studies published between January 1^th^ 2002 and March 23^th^ 2022 reporting incidence of SSI | Studies focusing on patients below the age of 18 |

Table 2 Table of primary and secondary outcomes of interest and inclusion and exclusion criteria
